# Electricity consumption dataset of a local energy cooperative

**DOI:** 10.1016/j.dib.2024.110373

**Published:** 2024-03-30

**Authors:** Francisco Monteiro, Rafael Oliveira, João Almeida, Pedro Gonçalves, Paulo Bartolomeu, Jorge Neto, Ricardo Deus

**Affiliations:** a Instituto de Telecomunicações, DETI - Universidade de Aveiro, Campus Universitário de Santiago, 3810-193 Aveiro, Portugal; b Digitalmente Lda., Rua Padre Donaciano Abreu Freire N° 43 R/C A, 3860-384 Estarreja Portugal; c Instituto de Telecomunicações, Universidade de Aveiro, Campus Universitário de Santiago, 3810-193 Aveiro, Portugal; d Instituto de Telecomunicações, ESTGA - Universidade de Aveiro, Campus Universitário de Santiago, 3810-193 Aveiro, Portugal; e Instituto Português do Mar e da Atmosfera, Rua C do Aeroporto, 1749-077 Lisboa, Portugal

**Keywords:** Local energy cooperative, Electricity consumption, Weather data, Energy community

## Abstract

Real-world data collections are generally not easily available. Energy measurements from buildings, houses and other devices can be used within different areas of research while being employed to plan or train models, allowing the improvement of power grid energy efficiency or providing more insight on how an energy community can work.

This paper provides a dataset concerning a Portuguese community of 172 households that are geographically close to each other, enabling the establishment of relationships among buildings and the analysis of a community's power consumption.

In addition to the consumed energy values, the related local weather information is included in the data. The intersection of weather data and energy measurements can be helpful to train AI models, contributing to explain variations in energy consumption and the absolute values of the energy readings.

The inclusion of these weather parameters aims to unveil features that can correlate to the energy measurements, enabling them to be used in multiple areas of research. Hence, it will provide added value to the data as it can be reused to explore Machine Learning algorithms or community energy planning by grid operators.

Specifications Table

**Table:** 

Subject	Electrical and Electronic Engineering
Specific subject area	The dataset contains information about the electrical consumption and meteorological data gathered from 172 residential buildings belonging to a local energy cooperative.
Data format	Raw
Type of data	Table, numbers
Data collection	The data was acquired using multiple smart meter devices communicating readings every 15 min to a central server. The measurements were collected between 05/05/2022 to 02/09/2023 in 172 different houses, located in the same parish (Loureiro, Oliveira de Azeméis) in the north of the Aveiro district, Portugal. All the collected data was included without any pre-processing.
Data source location	Loureiro, Oliveira de Azeméis, Portugal Latitude/Longitude: 40.80297356919648, −8.539664207470459
Data accessibility	Repository name: Electricity consumption dataset of a local energy cooperative Data identification number: 10.17632/vryvyfz2tj.1 Direct URL to data: https://data.mendeley.com/datasets/vryvyfz2tj/1 Instructions for accessing these data: Just access the link.

## 1 Value of the Data

• The dataset provides real-world energy collected data from buildings in a small geographic location and diverse weather measurements, allowing to characterize the electricity energy consumption profile for the region inhabitants, and to correlate consumption habits depending on weather conditions, such as temperature, light, or humidity.
• This particular type of dataset, with real-world energy collected data from multiple buildings in a small region, allows the construction of consumption forecast models free of effects related to local events, which in this case are common to all energy consumption locations.
• Since the European Union has established several policies to help the transaction of energy inside communities, it can help researchers or data scientists to test environments of energy communities where machine learning models and demand-side flexibility could be implemented. Also, it can be helpful to test both peer-to-peer and community-based models, since it aggregates a relatively small group of buildings.
• The dataset can be used by energy researchers, as well as energy managers, especially energy communications managers, in defining the terms of energy supply contracts, in community planning and to help understand consumption behaviors and trends. They can also be used by consumers when making decisions related to the purchase/sale/storage of energy, considering future consumption prospects and the weather conditions.
• The dataset can also be used by researchers in the construction of learning models, capable of predicting future consumption and models that can be improved by learning the impact caused by the related environment conditions.

## 2 Background

Cooperativa Eléctrica de Loureiro (CEL) is an electrical cooperative that purchases medium voltage electricity, transforms it and ensures the energy supply for the entire parish of Loureiro. The cooperative has focused on developing energy efficiency and renewable energy solutions. Among efforts to improve the energy efficiency of the cooperative and its members, the cooperative installed smart metering equipment, which measures and aggregates consumption data for 15 min intervals.

The Portuguese Institute for Sea and Atmosphere, I. P. (IPMA, IP), is a public institution, with responsibilities at national territory level in the areas of the sea and atmosphere. IPMA I. P. is a state laboratory and the national authority in the fields of meteorology, aeronautical meteorology, climate, seismology, and geomagnetism. It periodically monitors meteorological variables through a network with more than 130 automatic weather stations connected in near real time, continuously creating a record with aggregated data every 10 min. It also forecasts weather conditions for the entire country.

This dataset combines electricity consumption from the CEL cooperative members with meteorological data collected at the nearest IMPA station from Loureiro parish, for the same time interval. The main motivation behind the compilation of this dataset resides on the authors’ research interest to analyze electricity consumption in the context of a potential energy community, by employing machine learning models to predict future energy consumption by community members, thus enabling intelligent management decisions.

## 3 Data Description

The dataset was published as a CSV file that comprises the energy consumption measurements of 172 different buildings, that are part of a local cooperative, i.e., they are geographically close to each other. These measurements were collected from the buildings’ smart meters, in Loureiro, Portugal, and communicate every 15 min the amount of energy consumed in that period. The dataset includes 46,608 rows of 15 min intervals, and each column is related to one building's consumption values, besides the “Time” column and the meteorological data. The data is organized by date and time, and it covers the period between 05 and 05–2022 and 02–09–2023. Some buildings have entries missing, which are marked as Not a Number (NaN).

In addition to the energy data, related local weather information was added to complement the energy measurements. Both the energy and weather data were collected at the same time and have the same number of rows, with the respective dates. The weather data was taken from the nearest weather station to Loureiro, which is in Aveiro, Portugal (40°38′07.4″N 8°39′34.6″W).

To better understand the data structure, a detailed explanation of each column follows:

• “Time”, date and time of the recorded measurement
• “Energy_Meter_x”, energy consumption in 15-minute intervals (kWh) of the meter with ID x
• “Avg_Temp”, which is the average air temperature at 1.5 m above ground, presented in degrees Celsius (°C)
• “Avg_Rel_Humidity”, the average relative humidity in percentage
• “Avg_Wind_Direction”, which is the wind direction, from 0 to 360°
• “Avg_Wind_Speed”, which is the wind speed in m/s
• “Max_Inst_Wind_Speed”, which is the maximum instantaneous wind speed, also in m/s
• “Inst_Temp”, the instantaneous air temperature at 1.5 m, in degrees Celsius (°C)
• “Quantity_Precip”, which is the quantity of precipitation in millimeters
• “Max_Inst_Precip”, which is the maximum instantaneous precipitation intensity in mm/h
• “Total_Global_Rad”, which is the total global radiation, measured in KJ/m2

## 4 Experimental Design, Materials and Methods

Consumption data was collected from a local energy cooperative that encompasses over a hundred buildings in a small rural community. Each building is equipped with a meter responsible for recording electricity consumption. To streamline access to data from all meters in the area, multiple transformer posts are put in place, with each one being responsible for the meters of a specific area. These transformer posts aggregate several data types from all meters in the area, including readings and load curves, with a specific emphasis on the latter. Meters forward this information to the transformer post via a PRIME network using Power Line Communication (PLC) technology. While this data is accessible at the transformer post, there is also an option to upload the load curves to an FTP server, simplifying the information collection. Every day, the data is uploaded to the FTP server, regarding information for the current day. Once uploaded, software hosted on the server processes the load curves and inserts them into a relational database. Access to the recently processed information is available via an API, providing a complete platform for data retrieval and analysis.

### 4.1 Energy collection

**Fig. 1:**
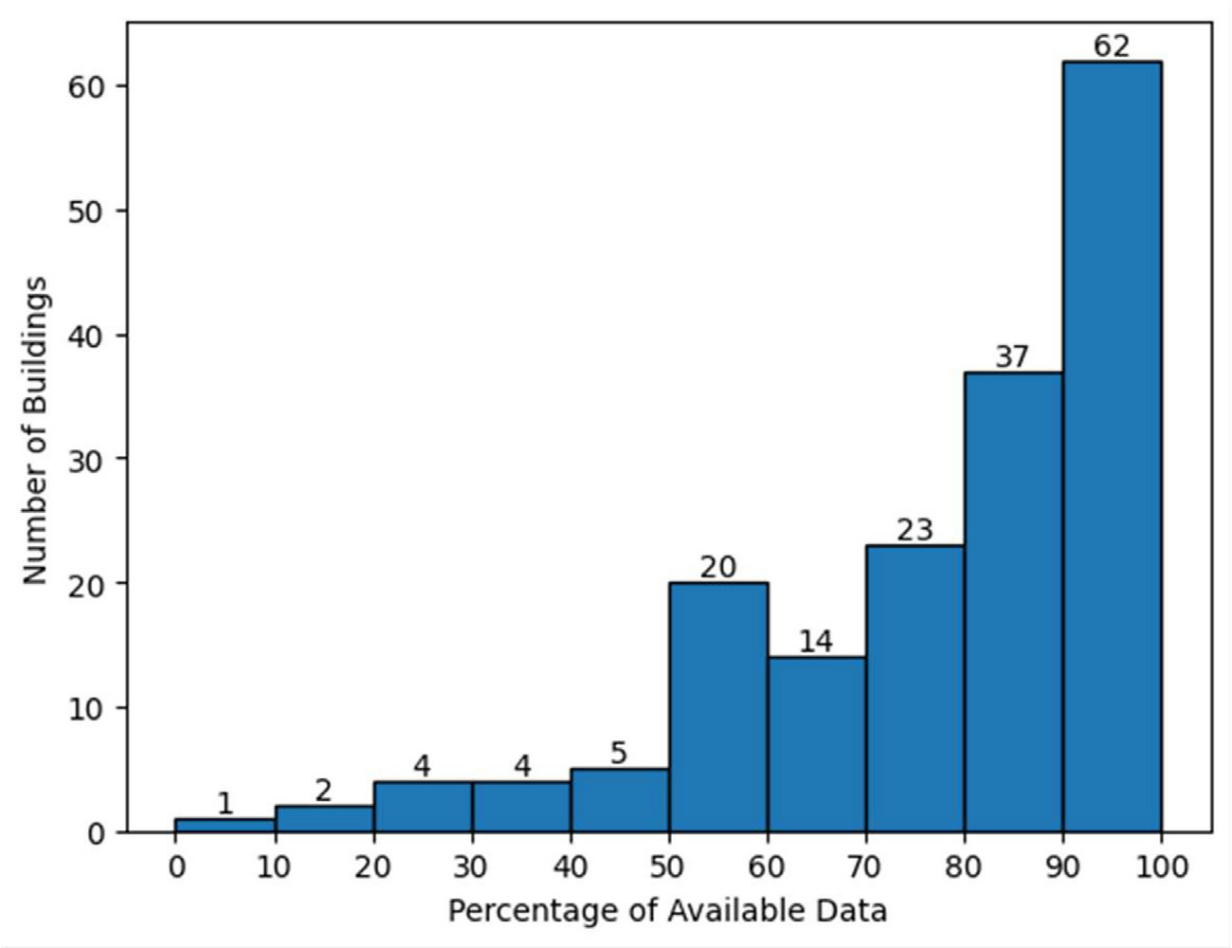
Number of buildings per data availability percentage range.

The entire community consists of 172 buildings that provided their energy consumption information. Regarding the electricity consumption values, no data processing such as data cleaning or outlier removal was performed, provided that the goal is to contribute a real dataset. In data processing tasks, while there may be a need for this, the decision was to entrust the selection of relevant data to the users of the dataset. By observing Fig. 1, it is possible to verify that there are 62 houses with at least 90 % of data available, which is more than 1 year of electricity readings.

### 4.2 Weather collection

The weather data was gathered by Instituto Português do Mar e da Atmosfera (IPMA) [1] at the Aveiro station [2]. The data was made available in intervals of 10 min. Hence, the data was resampled into 15 min intervals to match the energy consumption values. This resampling was done by averaging the 10th and 20th minutes of each hour and assigning it into the 15-minute interval of that hour, while also averaging the 40th and 50th minutes and assigning it into the 45 min interval of the same hour, maintaining the 0 minand the 30 min. values.

## Limitations

In some buildings, there are some missing values. These could not be retrieved due to technical problems in the smart meter data collection or due to power outages. Nevertheless, there are over 100 buildings with at least 35,000 records, which corresponds to approximately one year of electricity consumption metering.

## Ethics Statement

Authors have read and follow the ethical requirements for publication in Data in Brief and confirm that the current work does not involve human subjects, animal experiments, or any data collected from social media platforms. Table 1

**Table 1:** illustrates the structure of the dataset.

	Time	Energy_Meter _1	Energy_Meter_2	Energy_Meter_172	Avg_Temp	Avg_Rel_Humidity	Avg_Wind_Direction	Total_Global_Rad
0	2022–05–05 12:00:00	0.068	NaN	0.008	22.70	62.0	305.0	526.0
1	2022–05–05 12:15:00	0.070	NaN	0.056	22.55	63.5	311.5	522.35
2	2022–05–05 12:30:00	0.122	NaN	0.033	22.10	64.0	311.0	520.00
3	2022–05–05 12:45:00	0.132	NaN	0.062	21.95	62.5	300.0	514.40
4	2022–05–05 13:00:00	0.125	NaN	0.024	22.20	62.0	314.0	507.00
46,607	2023–09–02 23:45:00	0.140	0.069	0.034	19.45	74.5	36.5	0.00

## CRediT authorship contribution statement

**Francisco Monteiro:** Data curation, Writing – original draft, Visualization, Investigation, Software, Validation, Writing – review & editing. **Rafael Oliveira:** Data curation, Writing – original draft, Visualization, Investigation, Software, Validation, Writing – review & editing. **João Almeida:** Conceptualization, Methodology, Software, Data curation, Writing – original draft, Supervision, Writing – review & editing. **Pedro Gonçalves:** Conceptualization, Methodology, Software, Data curation, Writing – original draft, Writing – review & editing. **Paulo Bartolomeu:** Conceptualization, Methodology, Software, Data curation, Writing – original draft, Writing – review & editing. **Jorge Neto:** Conceptualization, Methodology, Software, Data curation, Writing – original draft, Visualization, Investigation, Supervision, Software, Validation, Writing – review & editing. **Ricardo Deus:** Conceptualization, Methodology, Software, Data curation, Writing – original draft, Visualization, Investigation, Supervision, Software, Validation, Writing – review & editing.

## Data Availability

Electricity consumption dataset of a local energy cooperative (Original data) (Mendeley Data).
